# Biomechanical properties of a novel fixation system for intra-articular distal humerus fractures: a finite element analysis

**DOI:** 10.1186/s13018-021-02836-1

**Published:** 2021-11-16

**Authors:** Lingpeng Kong, Yan Wang, Qingsen Lu, Yong Han, Fu Wang

**Affiliations:** 1 Department of Orthopaedics, Shandong Provincial Hospital Affiliated to Shandong First Medical University, No. 324, Jingwuweiqi Road, Jinan, 250021 Shandong People’s Republic of China; 2 Department of Medical Laboratory Diagnosis Center, Jinan Central Hospital, No. 105 Jiefang Road, Ji’nan, 250014 Shandong People’s Republic of China

**Keywords:** Distal humeral fracture, Capitellum, Trochlea, Biomechanics, Finite element analysis, Internal fixation

## Abstract

**Background:**

The traditional strategy for fixing intra-articular distal humerus fractures is double plating placed in an orthogonal configuration, based on posterior approach. With a combined medial and lateral approach, a novel configuration of plating (combined anteromedial and anterolateral plating) has been used. In this study, we investigated the biomechanical properties of the novel plating by comparing it with some traditional strategies.

**Methods:**

Based on the 3D morphology of a healthy subject’s humerus, models of three types of intra-articular distal humeral fractures were established using a variety of different internal fixation methods: (a) treatment of a simple intra-articular fracture of the distal humerus with the novel double plate and a traditional orthogonal plate; (b) treatment of a comminuted fracture of the lower distal humerus with the novel double plate, a traditional orthogonal plate and a traditional orthogonal plate combined with distally extended tension screws; (c) treatment of a coronal shear fracture of the distal humerus with the novel double plate, a traditional orthogonal plate and the intra-articular placement of three screws. The material properties of all plates and screws were isotropic and linearly elastic. The Poisson ratio of the implant and bone was 0.3, and the elastic modulus of the implant was 114,000 MPa. The axial loading is 200 N, the bending loading is 30 N and varus rotation is 7.5 Nm in the longitudinal direction.

**Results:**

A simple model of intra-articular fracture of the distal humerus (AO C1 type) was established. Under all experimental conditions, the novel double plate showed greater stiffness than the orthogonal double plate. The axial straightening, bending compression and varus torsion increased by 18.00%, 16.00% and 44.00%, respectively. In the model of comminuted fracture of the lower distal humerus, the novel double plate showed the best stiffness under three experimental conditions (163.93 N/mm, 37.97 N/mm, 2697.84 N mm/°), and the stiffness of the traditional orthogonal plate combined with the distally extended tension screws was similar to that of the traditional orthogonal plate (121.21 N/mm, 32.61 N/mm, 1968.50 N mm/°). In the model of coronal shear fracture of the distal humerus, the novel double plate showed the best stiffness under all test conditions (194.17 N/mm, 38.46 N/mm, 2929.69 N mm/°), followed by the traditional plate (153.85 N/mm, 33.33 N/mm, 2650.18 N mm/°), while the stiffness of the three screws was the smallest (115.61 N/mm, 28.30 N/mm, 2180.23 N mm/°).

**Conclusions:**

In terms of biomechanics, compared with other internal fixation methods, the novel combined anteromedial and anterolateral anatomical locking double-plate showed less stress, less displacement and greater stiffness. The novel double-plate method can be used to treat not only simple intra-articular fractures of the humerus but also complex comminuted fractures of the lower distal humerus and coronal shear fractures of the distal humerus, with a better effect than current traditional internal fixation methods.

## Introduction

Clinically, distal humeral fractures are difficult to treat, especially intra-articular fractures of the distal humerus, which are one of the most difficult fractures to treat [1]. The most effective treatment for intra-articular fractures of the distal humerus is open reduction and internal fixation (ORIF) [2]. When open reduction is performed, anatomical reconstruction of the articular surface of the distal humerus should be achieved. Internal fixation must be firm and stable, which is a key factor for good functional recovery after surgery [3]. Adequate exposure of the joint surface of the distal humerus is a prerequisite for good anatomical reduction and internal fixation [4]. A number of surgical approaches can be used to treat intra-articular fractures of the distal humerus to obtain good articular exposure, including the olecranon osteotomy approach [5], the triceps-reflecting anconeus pedicle (TRAP) approach [6], the triceps-splitting approach [7], the extensor mechanism-sparing paratricipital posterior approach [8], and the combined medial and lateral approach [9].

Because the olecranon osteotomy approach can fully expose the articular surface of the distal humerus, it is still the most commonly used surgical approach for intra-articular fractures of the distal humerus [10, 11]. However, it is also associated with many complications, such as bone nonunion, delayed union, heterotopic ossification, secondary surgery after internal fixation failure, and ulnar nerve palsy [11, 12]. Xie et al. [9] demonstrated that a medial and lateral approach to the elbow joint combined with medial and lateral incisions in the elbow joint could provide good exposure of the distal humeral articular surface and avoid olecranon osteotomy and ulnar nerve prepositioning, with fewer complications. Wei et al. [13] demonstrated that compared with the olecranon osteotomy approach, the approach consisting of combined medial and lateral incisions in the elbow joint mainly provided exposure of the humeral trochlea and capitellum in front of the distal humerus. For this approach, they designed a novel double-plate internal fixation method, with one plate placed on the anteromedial and the other on the anterolateral aspect. Finite element studies show that this novel fixation method was stronger than the orthogonal double-plate method and did not increase the stress, making it a feasible choice [14]. However, their combined anteromedial and anterolateral double plates were designed for the treatment of simple intra-articular fractures of the distal humerus. Complex comminuted fractures of the capitellum and trochlea and coronal shear fractures of the distal humerus were rarely involved. Fractures of the capitellum and trochlea of the humerus are most commonly displaced upward, and malunion can occur anterior to the distal humerus. This causes mechanical obstruction of the radial head and the trochlea when the elbow is bent, resulting in limited elbow movement. Furthermore, the displacement of fracture block, resulting in articular plane steps, can eventually lead to traumatic arthritis and severely impair elbow function [15]. Therefore, it is necessary to pay sufficient attention to this kind of injury and actively treat it in the clinic.

**Fig. 1:**
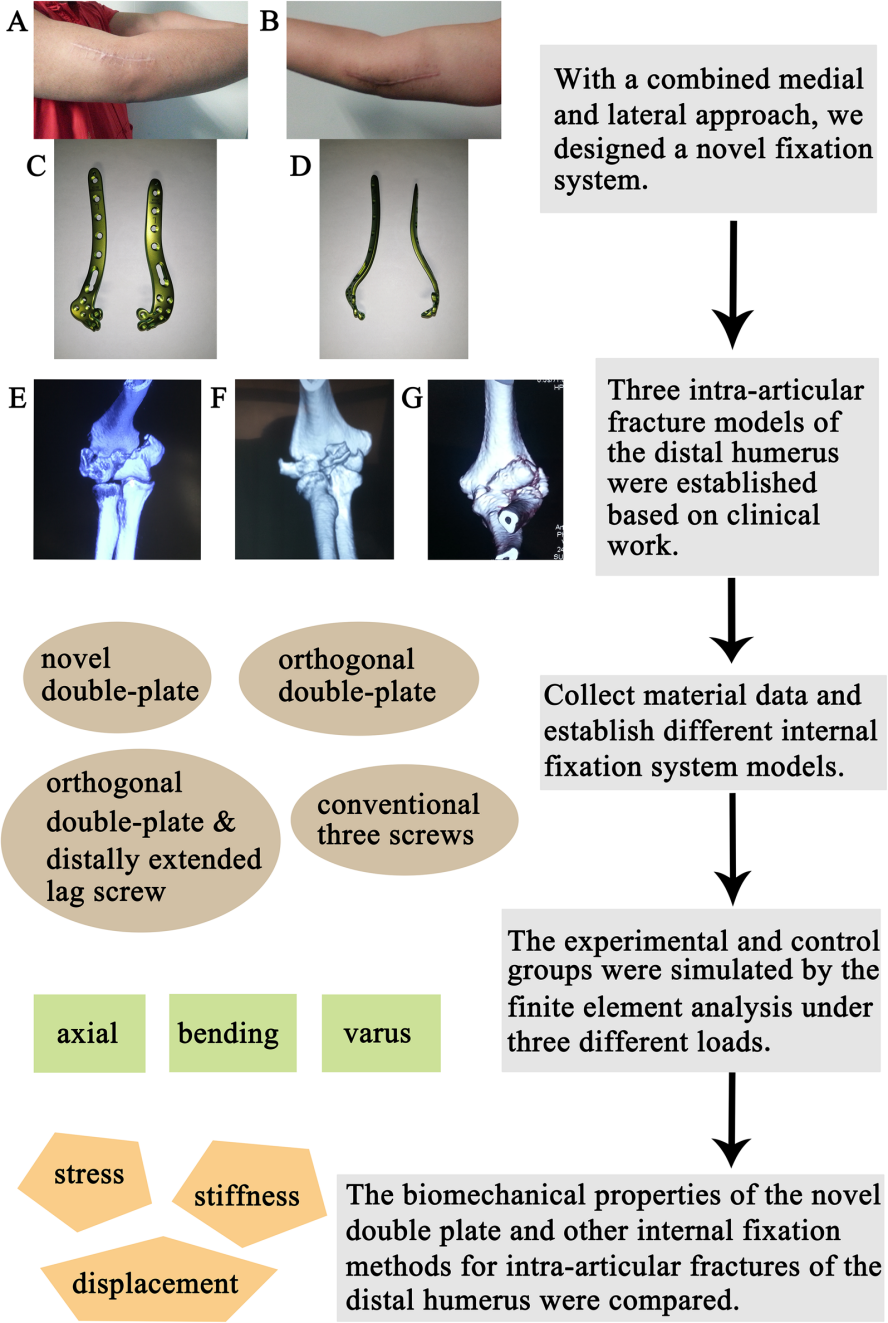
The concise experimental strategy of present study. **A**, **B** The combined medial and lateral approach. **C**, **D** A novel combined anteromedial and anterolateral anatomical locking double plate for fractures of the distal humerus designed by us. **E** Simple intra-articular fracture of the distal humerus (AO C1 type). **F** Comminuted fracture of the lower distal humerus. **G** Coronal shear fracture of the distal humerus

Due to the rarity of lower distal humeral fractures, there is still a lack of research on the specific clinical characteristics of and treatment strategies for lower distal humeral fractures. In particular, there is a lack of reports on the treatment of fractures of the capitellum with severe displacement of the trochlea and posterior condyle. Thus far, fractures of the capitellum, including coronal fractures of the humeral capitellum; fractures of the trochlea, including coronal fractures of the humeral trochlea; and intercondylar comminuted fractures of the humerus, have been without proper internal fixation instrumentation. A reconstruction plate is usually applied after bending, but it is difficult to bend, and the adhesion is poor; conventional locking plates are fixed with only one or two extended screws in the distal joint and cannot provide good treatment results. With a combined medial and lateral approach (Fig. 1A, B), we designed a novel combined anteromedial and anterolateral anatomical locking double plate for fractures of the distal humerus (Fig. 1C, D), in order to maximize the preservation and restoration of joint function and reduce complications and sequelae. The new plate not only holds well but also allows for more extended screws to be inserted at the distal end, thus providing better stability and greater fixation strength for more types of intra-articular fractures than conventional plates. In this study, the biomechanical properties of the novel double-plate method and other internal fixation methods were compared by finite element analysis. Firstly, three intra-articular fracture models of the distal humerus were established based on clinical work, including simple intra-articular fracture of the distal humerus (AO C1 type) (Fig. 1E), comminuted fracture of the lower distal humerus (Fig. 1F), and coronal shear fracture of the distal humerus (Fig. 1G). Secondly, we collected material data and establish different internal fixation system models, which mainly include novel double-plate, conventional orthogonal double-plate, orthogonal double-plate and distally extended lag screw and conventional three screws. Thirdly, the experimental and control groups were simulated by the finite element analysis under axial loading or anterior deflection, bending or posterior deflection and lateral or varus loading. Finally, the biomechanical properties (stress, displacement and stiffness) of the novel double plate and other internal fixation methods for intra-articular fractures of the distal humerus were compared. The results showed that the novel double plate can not only be used to treat simple intra-articular fractures but also be of great help in the treatment of complex comminuted fractures of the lower distal humerus and coronal shear fracture of the distal humerus.

## Materials and methods

### Research materials

This study was conducted at the Provincial Hospital Affiliated with Shandong First Medical University in Jinan, Shandong Province, China, with the permission of the hospital ethics committee. Before recruiting participants for this study, the authors obtained consent from the patients.

Computed tomography (CT) scans of implants were obtained (Siemens, Germany). The medial plate of the traditional orthogonal anatomical locking plate (Synthes, Shanghai, China) was 90 mm in length and 4.0 mm in thickness; the 4 proximal screws were 3.5 mm in diameter; the 2 distal screws were 2.7 mm in diameter. The dorsolateral plate was 120 mm in length and 4.0 mm in thickness; the 4 proximal screws were 3.5 mm in diameter; and the 5 distal screws were 2.7 mm in diameter. The novel anatomical locking double plate (Double, Xiamen, China) consisted of a medial plate 110 mm in length and 4.0 mm in thickness, with 4 proximal screws 3.5 mm in diameter and 3 distal screws 2.7 mm in diameter, as well as a lateral plate 90 mm in length and 4.0 mm in thickness, with 4 proximal screws 3.5 mm in diameter and 3 distal screws 2.7 mm in diameter.

### Establishment of an internal fixation model for intra-articular fractures of the distal humerus

The raw CT data of the volunteers and implants in the first part of the study were imported into Mimics Research 19.0 in DICOM format. The geometry was reconstructed using the Mimics (Materialise Belgium) and 3-matic Medical 12.0 (× 64) software packages.

**Fig. 2:**
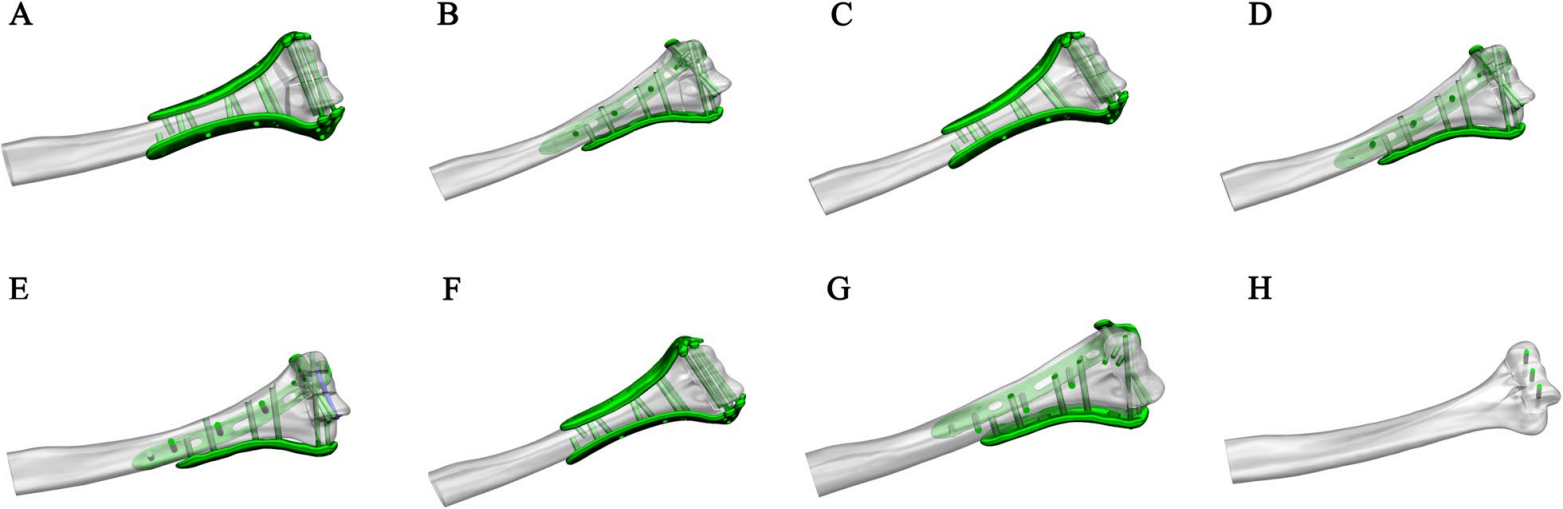
Models of intra-articular fractures of the distal humerus. **A** Novel double-plate fixation of a simple intra-articular distal humeral fracture. **B** conventional orthogonal double-plate fixation of a simple intra-articular distal humeral fractures. **C** Novel double-plate fixation of a distal humeral comminuted fracture. **D** Conventional orthogonal double-plate fixation of a distal humeral comminuted fracture. **E** Conventional orthogonal double-plate and distally extended lag screw fixation of a distal humeral comminuted fracture. **F** Novel double-plate fixation of a distal humeral coronal shear fracture. **G** Conventional orthogonal double-plate fixation of a distal humeral coronal shear fracture. **H** Fixation of a distal humeral coronal shear fracture with three screws

In this study, the team constructed three models of intra-articular distal humeral fractures. The novel anatomical locking double plate and conventional orthogonal anatomical locking plate and fully threaded screws were modelled. (1) Simple intra-articular fracture of the distal humerus (AO C1 type): One plate was placed anteromedial to the distal humerus, and the other was placed anterolaterally to establish a novel model of anatomical locking double-plate internal fixation (Fig. 2A). A traditional orthogonal double-plate model was established by placing one plate on the medial column and the other on the posterior aspect (Fig. 2B). (2) Comminuted fracture of the lower distal humerus: As shown in Fig. 2C, D, E, a fracture line was made along the upper edge of the trochlea and the capitellum, and then, the trochlea, the capitellum and part of the medial epicondylar were separated into four parts to form four similar fracture fragments, simulating comminuted fracture of the capitellum and trochlea. A plate was placed anteromedial to the lower distal humerus, and another was placed anterolaterally to establish a novel model of anatomical locking double-plate internal fixation (Fig. 2C). A traditional orthogonal double-plate model was established by placing one plate on the medial column and the other on the posterior aspect (Fig. 2D). Internal fixation with the traditional orthogonal dissection of the locking plate and distally lengthened lag screws (orthogonal plate and lag screws) was modelled (Fig. 2E). (3) Coronal shear fracture of the distal humerus (Dubberley classification type 3 fracture): One plate was placed anteromedial to the distal humerus, and another was placed anterolaterally to establish a novel model of anatomical locking double-plate internal fixation (Fig. 2F). A traditional orthogonal double-plate model was established by placing one plate on the medial column and the other on the posterior aspect (Fig. 2G). In addition, a group consisting of internal fixation with three screws was added (Fig. 2H).

The profile of the placed plate was moulded according to the shape of the distal humerus. The distal end of the plate was fixed with a fully threaded extension screw to ensure that the fracture was firmly fixed. The plate hole was left empty above or near the fracture line. The screw was perpendicular to the plate and was adjusted to the appropriate length to ensure that it break through the contralateral cortex. For the interaction, the contact area of the plate and the screw are connected to each other, and the screw is inserted into the bone.

**Fig. 3:**
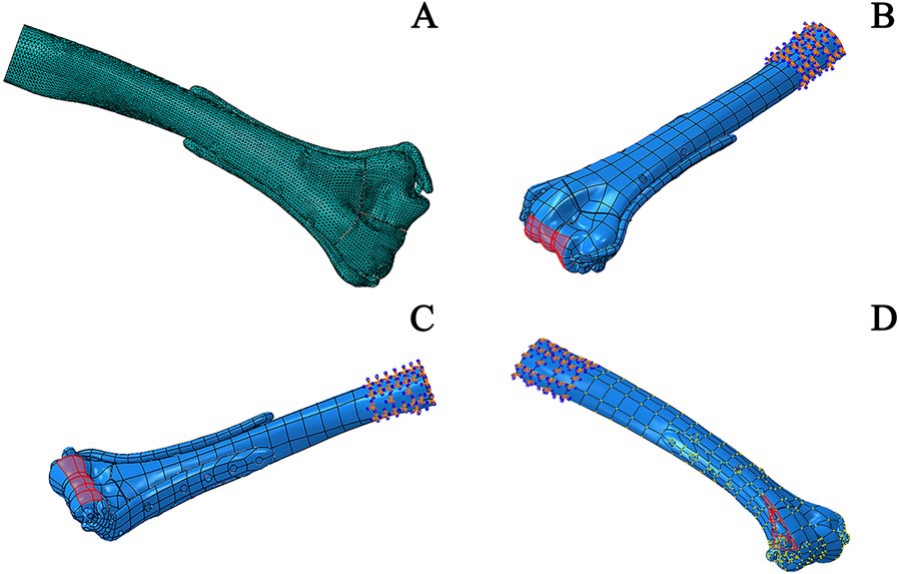
**A** Finite element meshing. **B** Axial compression load diagram. **C** Load diagram under bending test conditions. **D** Varus torsion load diagram

According to the bone segmentation function of Mimics 19.0 (Materialise, Belgium), mesh division of the model was performed using a 10-node quadratic tetrahedral element (C3D10) (Fig. 3A). To simplify the model, the threads of the locking screws were omitted. (1) Simple intra-articular fracture of the distal humerus (AO C1 type): The novel anatomical locking double-plate model contained a total of 178,577 elements and 255,958 nodes; the traditional orthogonal anatomical locking plate model contained a total of 199,884 elements and 286,339 nodes. (2) Comminuted fracture of the lower distal humerus: The novel anatomical locking double-plate model contained a total of 197,519 elements and 285,805 nodes; the traditional orthogonal anatomical locking plate model contained a total of 206,709 elements and 298,183 nodes; the distal humerus orthogonal plate and lag screw model contained a total of 203,846 elements and 293,907 nodes. (3) Coronal shear fracture of the distal humerus (Dubberley Classification type 3): The novel anatomical locking double-plate model contained a total of 251,342 elements and 362,897 nodes; the traditional orthogonal anatomical locking plate model contained a total of 203,262 elements and 291,668 nodes; the three-screw model contained a total of 106,228 elements and 140,629 nodes. Element sizes were selected based on mesh convergence analysis of displacement and von Mises stresses using a complete humeral model.

### Finite element biomechanical analysis

The experimental and control groups were simulated by the finite element method under axial, transverse and bending loads.

Finite element analysis was performed in Abaqus 6.14 (3DS, Waltham, MA). The finite element software ABAQUS 6.10-1 (Dassault Systemes, France) was used for the simulation. Each model was 180 mm long and limited at the proximal end. The material properties of all plates and screws were isotropic and linearly elastic. The Poisson ratio of the implant and bone was 0.3, and the elastic modulus of the implant was 114,000 MPa. Cortical bone and cancellous bone were distinguished. The elastic modulus of cancellous bone was 2000 MPa, and the elastic modulus of cortical bone was 3400 MPa for axial compression, 1150 MPa for post-flexion, and 660 MPa for varus loading [16–21]. In the three distal humeral fracture models, the proximal humerus was defined to be completely fixed on the X, Y and Z axes in the loading module of the software. The interaction relationship between the models of the humerus and plate was defined using the interaction module of the software. The contact interaction between the bone and plate was defined using surface-to-surface finite sliding with a coefficient of friction of 0.3 [22]. The contact relationship between the screw and plate and between the screw and surrounding bone was defined as binding. All contact elements were defined as deformable elements.

Based on a biomechanical study of distal humeral fractures [23–25], the total load was divided between the radial and ulnar columns at a ratio of 60–40% of the total load, respectively. The loading and position of the humerus were as follows [23, 24, 26]: axial loading or anterior deflection in which the upper arm was placed at a flexion angle of approximately 5° in relation to the longitudinal axis of the humeral diaphysis; bending or posterior deflection with the upper arm flexed at an angle of 75° in relation to the longitudinal axis of the humeral diaphysis; lateral or varus loading in which the load was applied laterally on the radial condylar surface, with the humerus placed horizontally. According to some literature reports [13, 18], plastic deformation of the model can be avoided when the axial loading is 200 N, the bending loading is 30 N and varus rotation is 7.5 Nm in the longitudinal direction. As shown in Fig. 3B–D, these loads were applied to the surface of the model.

### Evaluation criteria

ABAQUS 6.14 finite element software (Dassault Systemes Simulia Corp, USA) was used for static simulation. Different distal humeral fracture internal fixation methods were compared among the three fracture models under different mechanical loading conditions in terms of the following parameters: (1) the stress distribution of the internal fixation instrumentation, including the maximum stress; (2) the displacement distribution of the distal humeral fracture block; and (3) stiffness, which was calculated by dividing the load by the displacement or torque of the interaction point and of different units under compression and torsion conditions. Statistical methods were not used.

## Results

### Simple intra-articular fracture of the distal humerus (AO C1 type)

**Fig. 4:**
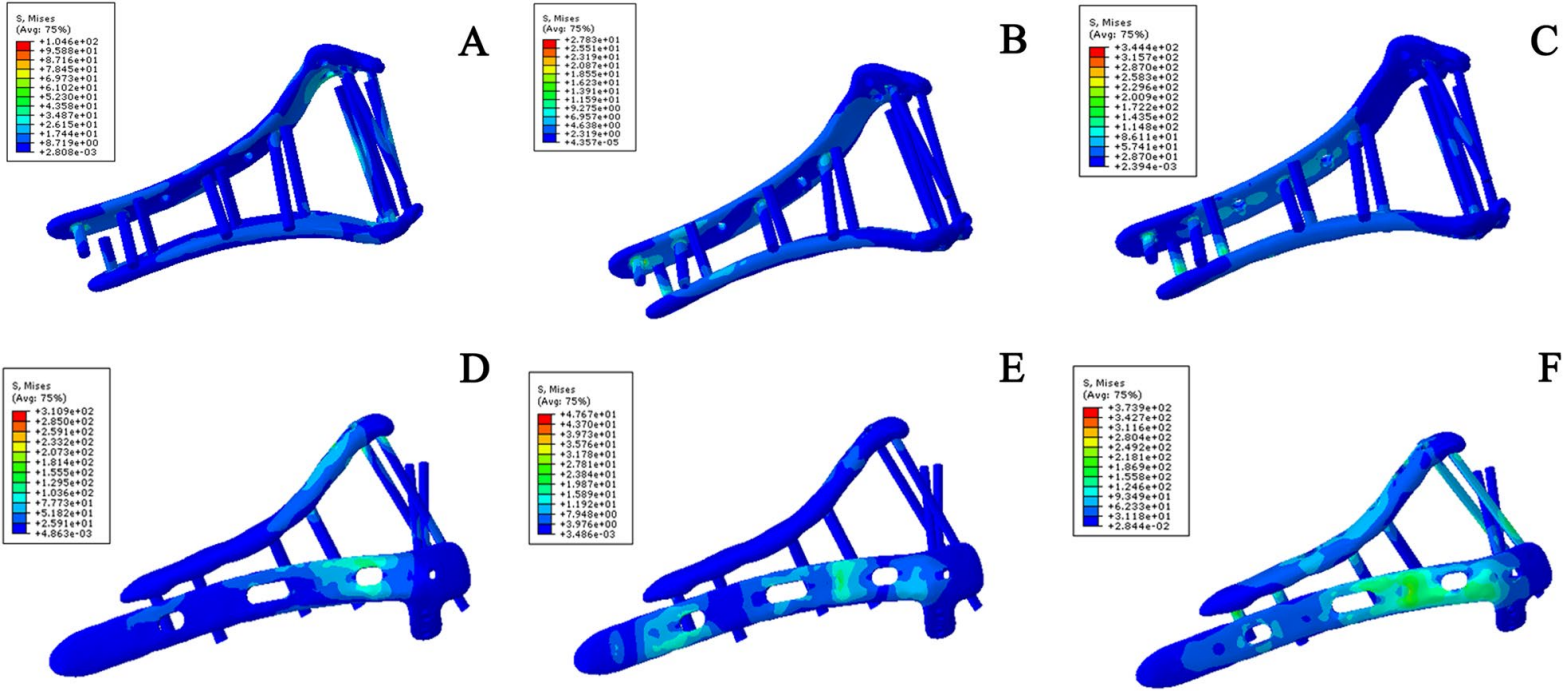
Maximum von Mises stress of the implant in a simple intra-articular fracture model of the distal humerus under different test conditions. **A** Stress of novel plating configuration under axial straightening. **B** Stress of novel plating configuration under bending compression. **C** Stress of novel plating configuration under varus torsion. **D** Stress of orthogonal plating configuration under axial straightening. **E** Stress of orthogonal plating configuration under bending compression. **F** Stress of orthogonal plating under varus torsion

Under the three test conditions, the influence of von Mises stress was weaker on the novel plate than the traditional orthogonal plate (Table 1). This shows that the novel plate could disperse the stress better in the treatment of simple intra-articular fractures of the distal humerus (AO C1 type) (Fig. 4).

**Table 1:** Maximal von Mises stresses of simple intra-articular fracture of the distal humerus models under different testing conditions

Configuration	Part	Axial (MPa)	Bending (MPa)	Rotational torsion (MPa)
Novel plating	Bone	9.67	4.70	39.25
	Plate and screw	104.60	27.83	344.40
Orthogonal plating	Bone	28.67	16.11	45.11
	Plate and screw	310.9	47.67	373.9

**Fig. 5:**
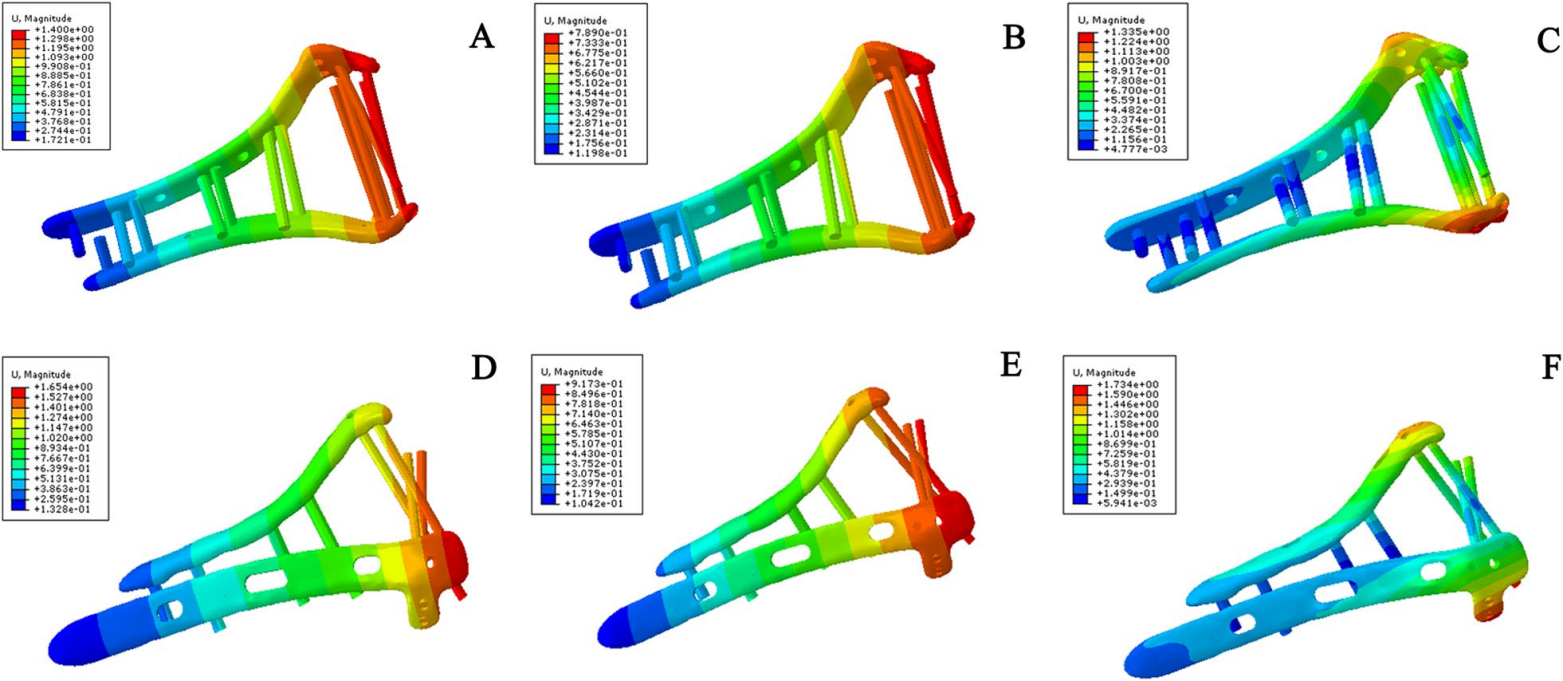
Implant displacement in a simple intra-articular model of the distal humerus under different test conditions. **A** Displacement of the novel plating configuration under axial straightening. **B** Stress of novel plating configuration under bending compression. **C** Stress of novel plating configuration under varus torsion. **D** Stress of orthogonal plating configuration under axial straightening. **E** Stress of orthogonal plating configuration under bending compression. **F** Stress of orthogonal plating configuration under varus torsion

Under the three test conditions, the displacement and rotational angle of the traditional orthogonal plate model were larger than those of the novel plate model (Fig. 5); the values are shown in Table 2. Similarly, under all experimental conditions, the stiffness of the novel double plate was greater than that of the orthogonal double plate. The axial straightening, bending compression and varus torsion increased by 18.00%, 16.00% and 44.00%, respectively (Table 3). The largest difference was found in internal rotation. This shows that the novel plate has greater stability, with the best resistance to varus torsion.

**Table 2:** Displacement of simple intra-articular fracture of the distal humerus models under different testing conditions

Configuration	Axial (mm)	Bending (mm)	Rotational torsion (°)
Novel plating	1.40	0.79	2.64
Orthogonal plating	1.65	0.92	3.81

**Table 3:** Stiffness of simple intra-articular fracture of the distal humerus models under different testing conditions

Configuration	Axial (N/mm)	Bending (N/mm)	Rotational torsion (N mm/°)
Novel plating	142.86	37.97	2840.91
Orthogonal plating	121.21	32.61	1968.50

### Comminuted fracture of the lower distal humerus

**Fig. 6:**
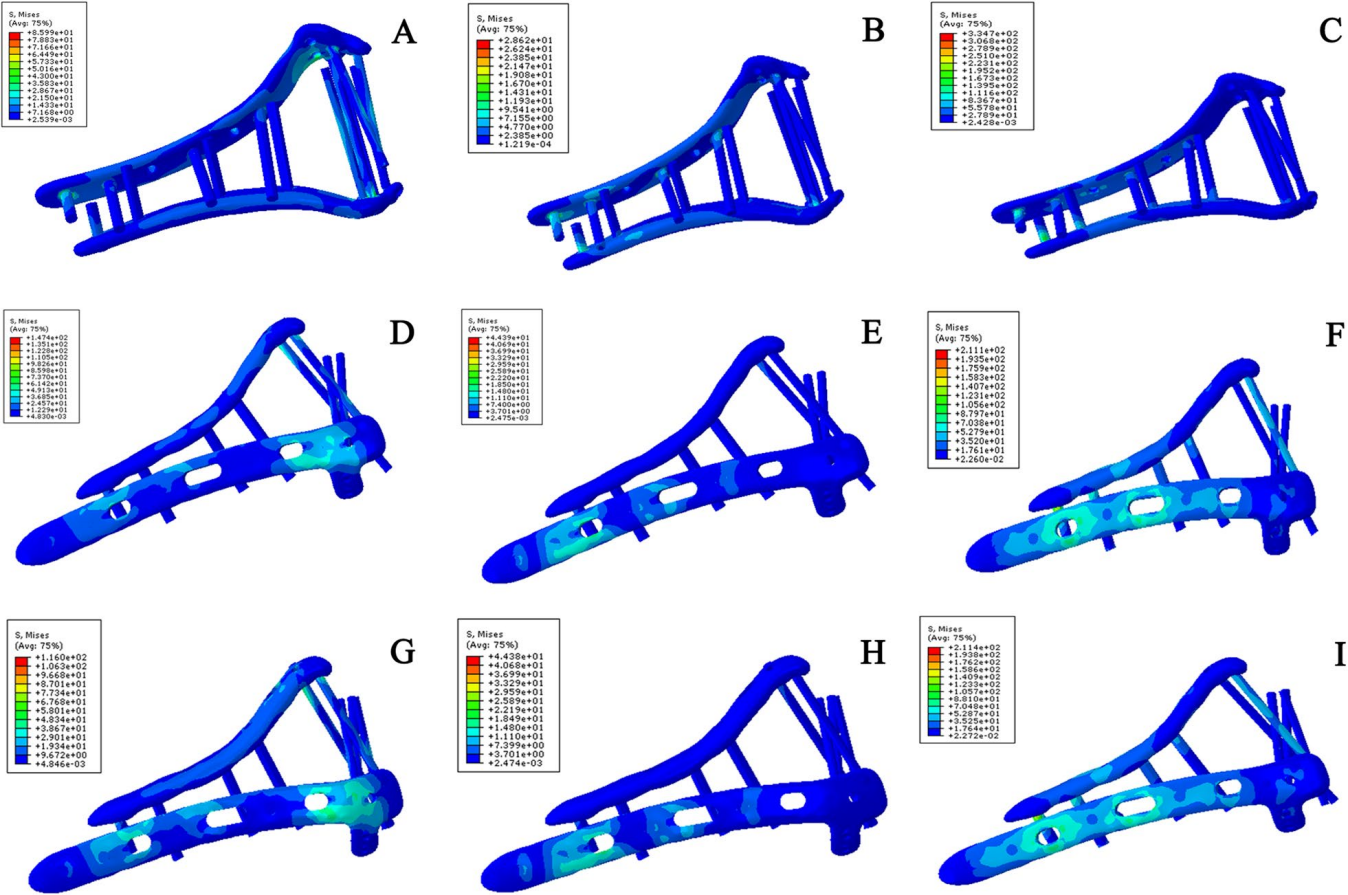
Maximum von Mises stress of the implant in a comminuted distal humeral fracture model under different test conditions. **A** Stress of novel plating configuration under axial straightening. **B** Stress of novel plating configuration under bending compression. **C** Stress of novel plating configuration under varus torsion. **D** Stress of orthogonal plating configuration under axial straightening. **E** Stress of orthogonal plating configuration under bending compression. **F** Stress of orthogonal plating configuration under varus torsion. **G** Stress of orthogonal plating and lag screw configuration under axial straightening. **H** Stress of orthogonal plating and lag screw configuration under bending compression. **I** Stress of orthogonal plating and lag screw configuration under varus torsion

Under the three test conditions, compared with the other two internal fixation methods, the novel double-plate method showed lower von Mises stress (Table 4), demonstrating a certain advantage over traditional internal fixation in terms of stress dispersion in the treatment of comminuted fractures of the lower distal humerus (Fig. 6). At the same time, under the axial compression condition, the stress of fixation with orthogonal plates and lag screws was lower than that of orthogonal plates, but under the other two test conditions, the stress of both internal fixation methods was similar. This shows that for comminuted fractures of the lower distal humerus, fixation with orthogonal plates and lag screws could disperse the stress better than that with orthogonal plates under axial compression.

**Table 4:** Maximal von Mises stresses of comminuted fracture of the distal humerus models under different testing conditions

Configuration	Part	Axial (MPa)	Bending (MPa)	Rotational torsion (MPa)
Novel plating	Bone	12.74	5.93	102.60
	Plate and screw	85.99	28.62	334.70
Orthogonal plating	Bone	42.66	6.08	60.26
	Plate and screw	147.40	44.39	211.10
Orthogonal plating with lag screws	Bone	36.73	6.87	53.49
	Plate and screw	116.0	44.38	211.40

**Fig. 7:**
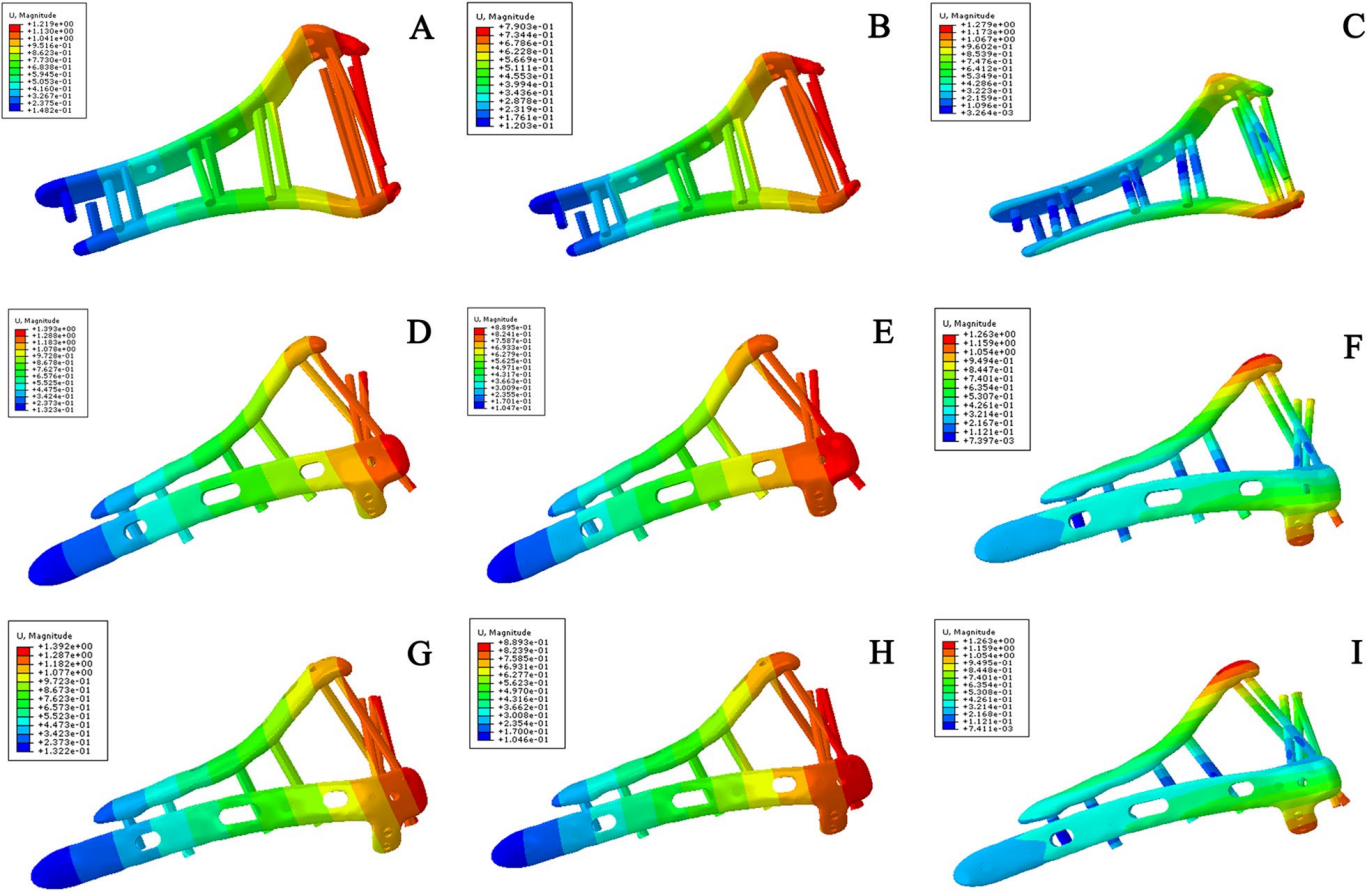
Implant displacement in a comminuted distal humeral fracture model under different test conditions. **A** Displacement of novel plating configuration under axial straightening. **B** Displacement of novel plating configuration under bending compression. **C** Displacement of novel plating configuration under varus torsion. **D** Displacement of orthogonal plating configuration under axial straightening. **E** Displacement of orthogonal plating configuration under bending compression. **F** Displacement of orthogonal plating configuration under varus torsion. **G** Displacement of orthogonal plating and lag screw configuration under axial straightening. **H** Displacement of orthogonal plating and lag screw configuration under bending compression. **I** Displacement of orthogonal plating and lag screw under varus torsion

Under the three test conditions, the displacement and rotational angle of the novel plate model were smaller (Fig. 7), while the displacement of the orthogonal plate and lag screw model was similar to that of the orthogonal plate model. The values are shown in Table 5. Under all experimental conditions, the stiffness of the novel plate model was the best, and the stiffness of the orthogonal plate and lag screw model was similar to that of the traditional orthogonal plate model (Table 6).

**Table 5:** Displacement of comminuted fracture of the distal humerus models under different testing conditions

Configuration	Axial (mm)	Bending (mm)	Rotational torsion (°)
Novel plating	1.22	0.79	2.78
Orthogonal plating	1.39	0.89	3.14
Orthogonal plating with lag screws	1.39	0.89	3.14

**Table 6:** Stiffness of comminuted fracture of the distal humerus models under different testing conditions

Configuration	Axial (N/mm)	Bending (N/mm)	Rotational torsion (N mm/°)
Novel plating	163.93	37.97	2697.84
Orthogonal plating	143.89	33.71	2388.54
Orthogonal plating with lag screws	143.89	33.71	2388.54

### Coronal shear fracture of the distal humerus

**Fig. 8:**
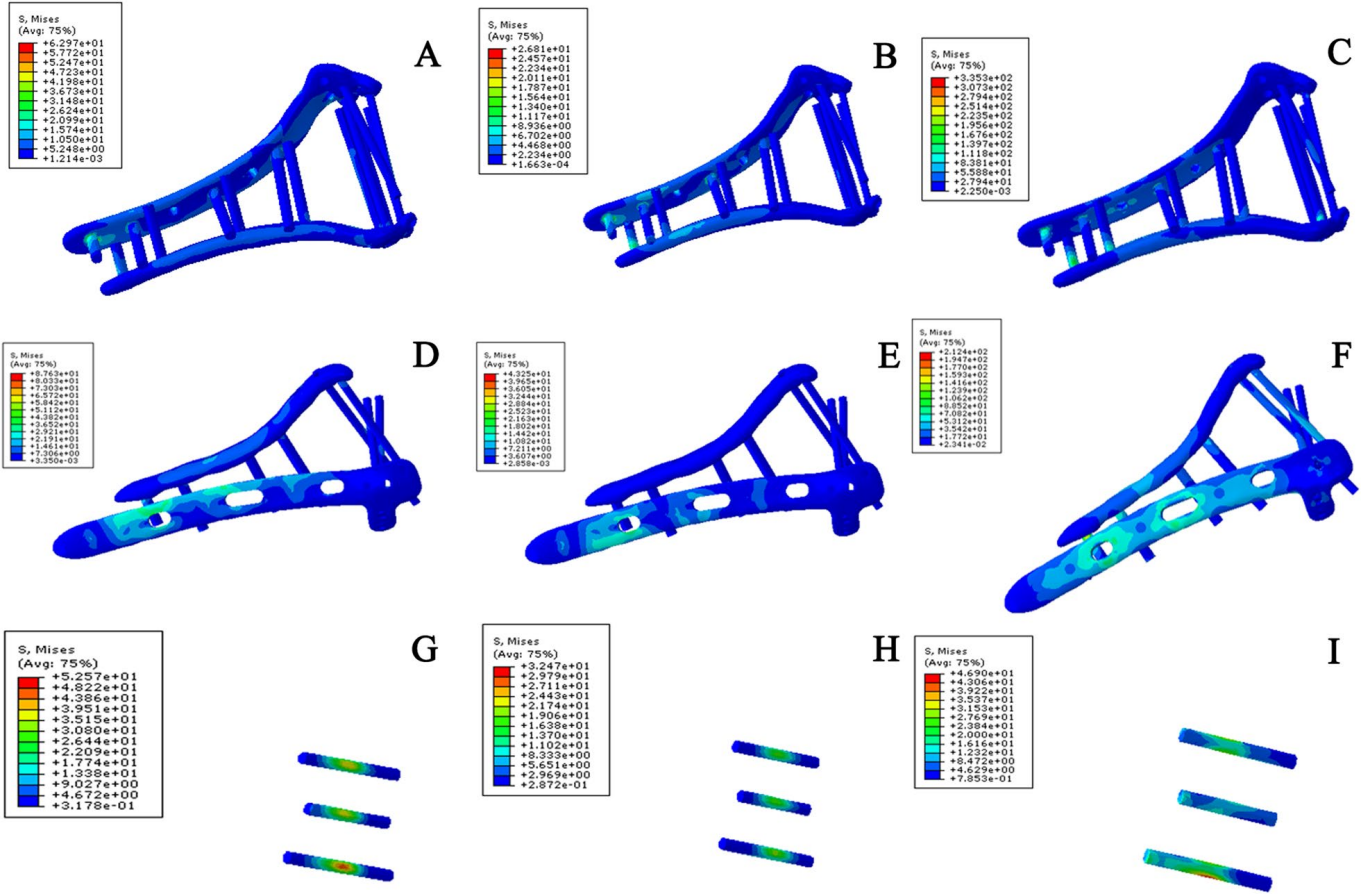
Maximum von Mises stress of the implant in coronal shear fracture of the distal humerus models under different test conditions. **A** Stress of novel plating configuration under axial straightening. **B** Stress of novel plating configuration under bending compression. **C** Stress of novel plating configuration under varus torsion. **D** Stress of orthogonal plating configuration under axial straightening. **E** Stress of orthogonal plating configuration under bending compression. **F** Stress of orthogonal plating configuration under varus torsion. **G** Stress of three-screw configuration under axial straightening. **H** Stress of three-screw configuration under bending compression. **I** Stress of three-screw configuration under varus torsion. The results show that for coronal shear fractures of the distal humerus, the three-screw fixation method has a certain advantage over the other two internal fixation methods in terms of stress dispersion

Under the condition of axial compression, the von Mises stress of the traditional orthogonal plate model was the highest, followed by that of the novel plate model and the three-screw model. Under the condition of bending compression, the von Mises stress of the traditional orthogonal plate model was the highest and that of the novel plate model was the smallest. Under the condition of rotational torsion, the von Mises stress of the novel plate model was the largest, followed by that of the traditional orthogonal plate model and the three-screw model (Table 7). This shows that the novel plating configuration has a certain advantage over orthogonal plating configurations in terms of stress dispersion in the treatment of comminuted fractures of the distal humerus (Fig. 8). Additionally, for coronal shear fractures of the distal humerus, the three-screw fixation method has a certain advantage over the other two internal fixation methods in terms of stress dispersion.

**Table 7:** Maximal von Mises stresses of coronal shear fracture of the distal humerus models under different testing conditions

Configuration	Part	Axial (MPa)	Bending (MPa)	Rotational torsion (MPa)
Novel plating	Bone	23.52	5.71	48.36
	Plate and screw	62.97	26.81	335.30
Orthogonal plating	Bone	11.87	7.06	45.47
	Plate and screw	87.63	43.25	212.40
Three screws	Bone	11.84	6.10	47.07
	Plate and screw	52.57	32.47	46.90

**Fig. 9:**
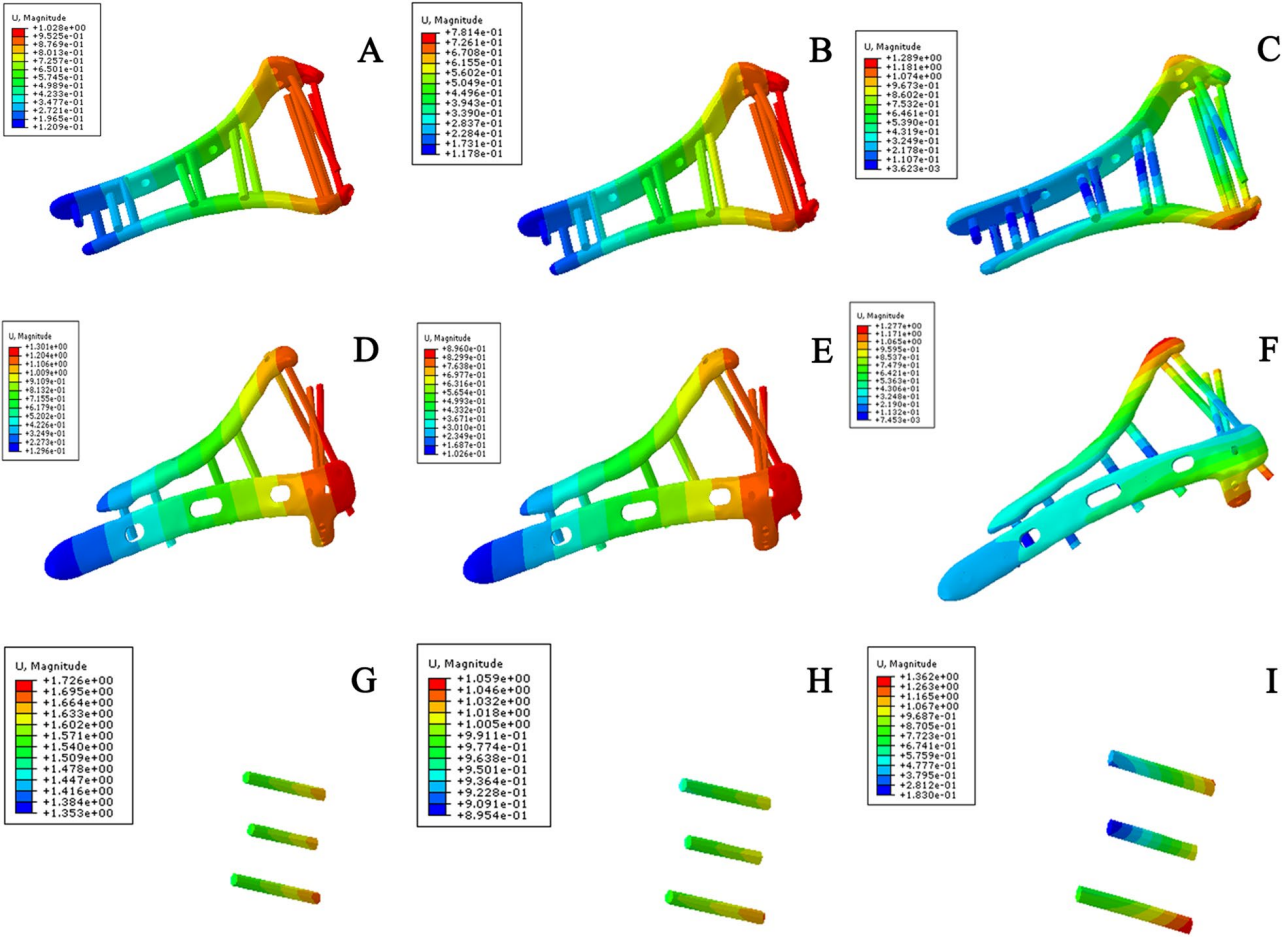
Implant displacement in coronal shear fracture of the distal humerus models under different test conditions. **A** Displacement of novel plating configuration under axial straightening. **B** Displacement of novel plating configuration under bending compression. **C** Displacement of novel plating configuration under varus torsion. **D** Displacement of orthogonal plating configuration under axial straightening. **E** Displacement of orthogonal plating configuration under bending compression. **F** Displacement of orthogonal plating configuration under varus torsion. **G** Displacement of three-screw configuration under axial straightening. **H** Displacement of three-screw configuration under bending compression. **I** Displacement of three-screw configuration under varus torsion. Under all the test conditions, the displacement and rotational angle of the novel plating model were smallest, followed by those of the traditional orthogonal plate model, while the displacement and rotational angle of the three-screw fixation model were the largest

Under all the test conditions, the displacement and rotational angle of the novel plating configuration were smaller (Fig. 9) than those of the traditional orthogonal plating configuration, while the displacement and rotational angle of the three-screw configuration were the largest, as shown in Table 8. Similarly, under all experimental conditions, the stiffness of the novel double-plate method was the best, followed by that of the traditional plate method, while the stiffness of the three-screw fixation method was the smallest (Table 9).

**Table 8:** Displacement of coronal shear fracture of the distal humerus models under different testing conditions

Configuration	Axial (mm)	Bending (mm)	Rotational torsion (°)
Novel plating	1.03	0.78	2.56
Orthogonal plating	1.30	0.90	2.83
Three screws	1.73	1.06	3.44

**Table 9:** Stiffness of coronal shear fracture of the distal humerus models under different testing conditions

Configuration	Axial (N/mm)	Bending (N/mm)	Rotational torsion (N mm/°)
Novel plating	194.17	38.46	2929.69
Orthogonal plating	153.85	33.33	2650.18
Three screws	115.61	28.30	2180.23

## Discussion

Distal humeral fractures account for approximately 15% of intra-articular fractures, and distal humerus coronal plane fractures account for approximately 80% of fractures of the capitellum and trochlea [15, 27, 28]. Orthogonal double-plate internal fixation is often preferred for the treatment of intra-articular fractures of the distal humerus [29] because it is generally considered to be the strongest form of internal fixation [30]. In clinical work, we found that it was difficult to achieve good results using traditional plates for the fixation of complex distal humeral fractures, such as coronal fractures of the capitellum and trochlea and distal humeral low comminuted fractures. A combined medial and lateral approach to the elbow exposes the anterior articular surface of the trochlea and capitellum. The novel plate is designed for use in a combined medial and lateral approach to the elbow, providing not only anteromedial and anterolateral support but also more extended screw placement at the distal end to better fix the intra-articular fracture. For coronal fractures, anterior fractures, and unilateral fractures, this method can provide greater stability and fixation strength than conventional fixation with posterolateral and medial plates. In this study, medical imaging and computer-aided virtual modelling software were used to establish the geometrical shape and material mechanical properties of the implants (plates and screws) and bone. The biomechanical advantages of the novel plating configuration under multiple loads were verified by finite element analysis.

Finite element analysis is a very valuable test method that is equivalent to experimental biomechanical research [20]. It can be used to solve a wide variety of problems in the fields of mathematics, physics, engineering, biology and orthopaedics [8]. Using finite element analysis, Wei et al. [13] showed that for simple intra-articular humeral fractures, combined anteromedial and anterolateral reconstruction with double plates resulted in less displacement and greater stiffness during axial compression, varus torsion, bending torsion and external rotational torsion than internal fixation with orthogonal double plates, with the greatest improvement in stiffness against varus torsion. In this study, loads were applied in the position of humeral flexion (75°) and extension (5°) with regard to the longitudinal humeral axis. The applied loads were within the physiological limits of loads in everyday activity during the postoperative rehabilitation period, and we avoided applying loads that would cause permanent plastic deformation of the implants. In addition to bending and axial loading, special attention was given to lateral or varus loading, as a previously neglected type of loading with pronounced clinical significance. Namely, the force of gravity, which acts on the long lever arm (the forearm) while the elbow is flexed and extended during activities apparently requiring minimal use, leads to repeated varus stresses in the elbow. As studied by O'Driscoll SW [31], pseudoarthrosis of the distal humerus usually occurs in the region at the metaphyseal and supracondylar level of the radial column due to varus torsion.

In the simulated biomechanical study, the novel double-plate internal fixation method showed greater stiffness. The main body of the plate and the screws are the key parts of the overall stability. The placement of screws across the fracture line can integrate adjacent fracture fragments and fix them together. Through the combined use of the screws and plate, the implant can fix all the fragments to the plate and then fix the plate to the humeral axis to form a relatively rigid structure. The profile of the bending part of the plate is consistent with the shape of the distal humerus, and this part is a weak link of stress conduction. The change in displacement was opposite to the change in stiffness, so the displacement data also prove the superior stability of the whole configuration. Compared with the traditional orthogonal plate, in the simple intra-articular fracture model of the distal humerus, the novel double plate showed obviously smaller effects of stress. This means that the novel double plate is effective in distributing high loads of stress. Under all the test conditions, the novel double plate showed less displacement and greater stiffness, which indicates that the novel double plate has more biomechanical advantages in the treatment of simple intra-articular fractures of the distal humerus (AO C1 type). In the comminuted fracture of the lower distal humerus model, including lengthened screws in fixation with the traditional orthogonal plate reduced the axial compressive stress of the traditional orthogonal plate. However, there was no obvious advantage in the stress under the other test conditions or in terms of displacement, rotational angle or stiffness. On the other hand, the novel double plate showed less displacement, a smaller rotational angle and greater stiffness under all loading conditions. This proves that the novel plating configuration has a better biomechanical effect than traditional configuration for comminuted fractures of the distal humerus. In the coronal shear fracture of the distal humerus model, fixation with three screws showed certain advantages over the other two internal fixation methods in terms of stress dispersion. Under all the test conditions, the displacement and rotational angle of the novel plating model were the smallest, and the stiffness was the best, which indicates that the novel plating configuration has excellent stability in the treatment of coronal shear fractures of the distal humerus.

The optimal approach for treating lower distal humeral comminuted fractures and coronal shear fractures has not yet been determined because they are complex intra-articular fractures with a low incidence [32]. Because the trochlea and olecranon form a semi-enclosed joint without soft tissue attachment and protection, lower distal humeral comminuted fractures and coronal shear fractures of the distal humerus are often displaced, with free fragments in the articular cavity. If left untreated, displacement and necrosis are likely to occur, leading to a poor clinical prognosis [33]. To better treat comminuted fractures of the lower distal humerus and coronal shear fractures of the distal humerus, we designed a novel combined anteromedial and anterolateral anatomical locking double plate. In this study, we investigated the biomechanical properties of this novel fixation method for the treatment of distal humeral intra-articular fractures by finite element analysis and compared it with other internal fixation methods for the treatment of comminuted fractures of the distal humerus and coronal shear fractures of the distal humerus. The study showed that the novel plating configuration has biomechanical advantages, with less stress, less displacement and greater stiffness, and is a better internal fixation method for lower distal humeral comminuted fractures and coronal shear fractures of the distal humerus.

In addition to suitable biomechanics, the novel double-plate internal fixation method has shown feasibility for use in clinical practice. First, the anteromedial and anterolateral sides of the distal humerus provide a relatively flat attachment condition for the plate. Second, due to the protrusion of the internal epicondyle of the humerus, it is difficult to pass the screw transversally through the capitellum to the trochlea when using a traditional orthogonal plate. Screws coming from the medial side, more often than not, proceed dorsolaterally and end between the medial and lateral columns, with only partial holding of the capitellum and trochlea; thus, the extra use of lag screws would sometimes be necessary. Notably, the lateral epicondyle curves more slightly than the medial epicondyle. The lateral plate fits the shape of the lateral column and can be placed distally more easily. Moreover, the novel double-plate internal fixation method includes more distal transverse screws than the traditional orthogonal double-plate internal fixation method, which allows for more solid intra-articular fixation of distal humeral fractures, in turn allowing early postoperative exercise and promoting rapid postoperative recovery.

The novel combined anteromedial and anterolateral anatomical locking double-plate has not been used in clinical practice, and the finite element analysis in this study has some limitations. First, the study did not use a large sample size but only a humerus with a standard morphology. Second, this study did not consider factors such as soft tissue insertion and bone destruction, so the results may overestimate the stiffness of internal fixation. Third, the study did not simulate other methods of internal fixation, such as lag screws, headless screws, and Kirschner wires, which could complicate the analysis and make the results less reliable.

## Conclusion

When used for the treatment of simple intra-articular fractures of distal humerus, comminuted fractures of the lower distal humerus and coronal shear fractures of the distal humerus, in terms of biomechanics, the novel combined anteromedial and anterolateral anatomical locking double-plate fixation method exhibited less stress, less displacement and greater stiffness than other internal fixation modalities. The novel double-plate can be used not only to treat simple intra-articular fractures of the humerus but also to treat complex comminuted fractures of the lower distal humerus and coronal shear fractures of the distal humerus and has a better effect than current traditional internal fixation methods. In the future, after further biomechanical and clinical studies, this method may be widely used in the treatment of intra-articular fractures of the distal humerus.

## Data Availability

All data analysed during this study are included in this published article.

## Abbreviations

CT: Computed tomography
